# Underwater endoscopic submucosal dissection using conical hood and gel immersion for esophageal squamous cell carcinoma with anastomotic stricture after total pharyngolaryngectomy

**DOI:** 10.1055/a-2493-8400

**Published:** 2024-12-12

**Authors:** Takahiro Muramatsu, Masakatsu Fukuzawa, Midori Mizumachi, Satoshi Shimai, Miki Wada, Manami Kajiwara, Takao Itoi

**Affiliations:** 1 38548 Department of Gastroenterology and Hepatology, Tokyo Medical University Hospital, Tokyo, Japan; 2 38548 Department of Diagnostic Pathology, Tokyo Medical University Hospital, Tokyo, Japan

Pharyngoesophageal defects after total pharyngolaryngectomy (TPL) are commonly reconstructed with free jejunum or anterolateral thigh flap (ALT), often resulting in anastomotic stricture 1 . Endoscopic treatment of superficial esophageal squamous cell carcinoma (ESCC) in the presence of such an anastomotic stricture is challenging and requires ingenuity of devices and scopes 2 . Endoscopic submucosal dissection (ESD) with water or gel immersion helps in difficult-to-treat situations 3 4 , and the utility of a small-caliber tapered conical hood during ESD is established 5 . Herein, we describe underwater ESD with a conical hood and gel immersion, which was performed successfully for superficial ESCC with post-TPL anastomotic stricture ( Video 1 ).

Underwater endoscopic submucosal dissection using conical hood and gel immersion for esophageal squamous cell carcinoma with anastomotic stricture after total pharyngolaryngectomy.Video 1

A 59-year-old woman with a history of TPL and ALT reconstruction for hypopharyngeal cancer presented with ESCC (20 mm, type 0-IIc) distal to the anastomotic stricture ( Fig. 1 ). The scope maneuverability was poor due to limited mouth opening, and the anastomotic stricture resulted in resistance to scope passage. ESD was attempted using a super-soft hood (Space Adjuster; TOP Corporation, Tokyo, Japan). However, the stricture could not be passed. Therefore, we used a small-caliber tapered conical hood (CAST hood; TOP Corporation, Tokyo, Japan) to enable passage of the stricture ( Fig. 2 **a–c** ). Underwater ESD was performed because of the poor scope maneuverability. As the visual field became obscured by hemorrhage and mucus during mucosal incision, gel (Viscoclear; Otsuka Pharmaceutical Factory, Tokushima, Japan) was added, and thus a clear view was obtained ( Fig. 2 **d–j** ). The underwater condition and the conical hood allowed an easy approach to the submucosal layer, resulting in successful en bloc resection ( Fig. 2 **k, l** ). Histopathological analysis revealed curative resection ( Fig. 3 ).

**Fig. 1:**
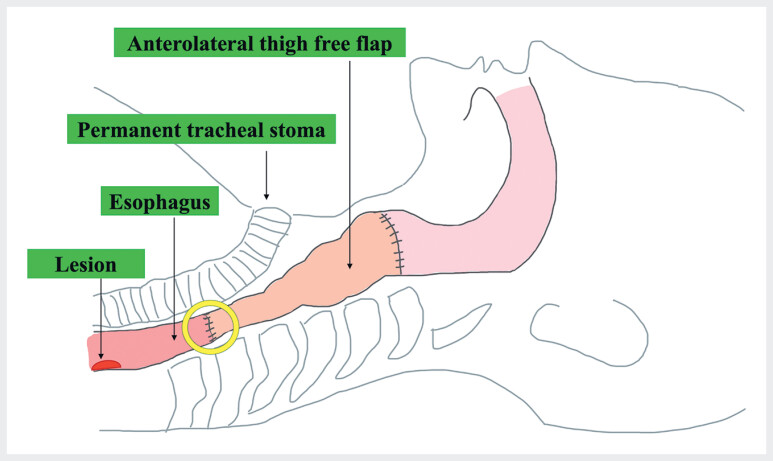
Schema of reconstruction using an anterolateral thigh flap for pharyngoesophageal defect after total pharyngolaryngectomy. Our patient had previously undergone this reconstruction procedure. A stricture can be observed at the distal end of the anastomosis (yellow circle), and beyond it the location of the superficial esophageal squamous cell carcinoma.

**Fig. 2:**
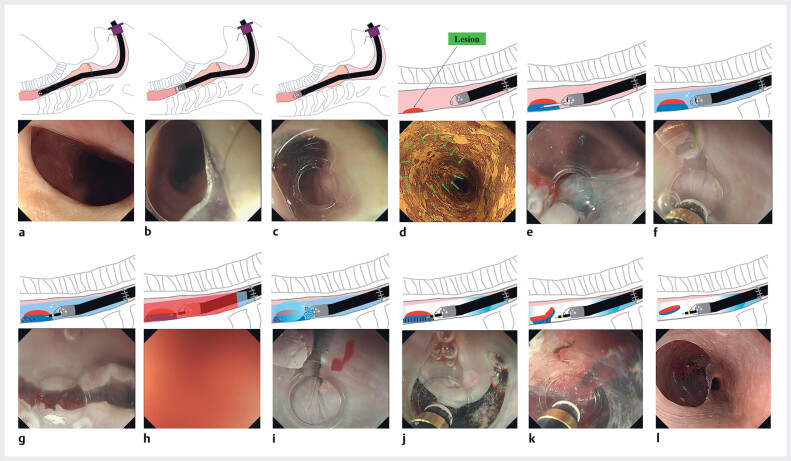
Schemas and endoscopic images of underwater endoscopic submucosal dissection using a conical hood and gel immersion. **a** Anastomotic stricture. **b** With the super-soft hood attached, the endoscope cannot pass through the stricture. **c** With the small-caliber tapered hood attached, the endoscope passes through the stricture. **d** The lesion is on the esophagus distal to the anastomotic stricture; after iodine staining it remains unstained (green arrows). **e** Local injection. **f** Underwater view. **g** A mucosal incision is made on the distal edge of the lesion for the endpoint. **h** The endoscopic view is poor due to bleeding and mucus. **i** Gel immersion provides a clear view. **j** A mucosal incision is made on the proximal side with water and gel immersion. **k** Submucosal dissection is performed. **l** Complete en bloc resection is achieved.

**Fig. 3:**
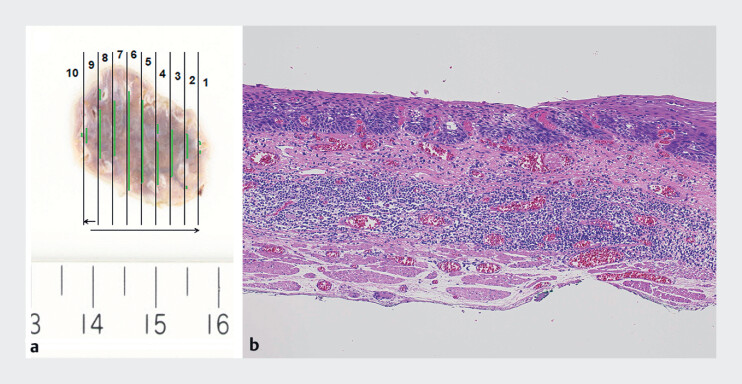
Macroscopic and histopathological images of the resected specimen. **a** Macroscopic image of the specimen. **b** Histopathological image of the specimen. The pathological diagnosis was esophageal squamous cell carcinoma in the lamina propria mucosae with no lymphovascular invasion and negative margins.

In conclusion, when ESD is performed for ESCC in the presence of an anastomotic stenosis after TPL, underwater ESD technique using a conical hood and gel immersion can enable passage through the stricture and improve scope operability and the visual field, enabling safe resection under low pressure.

Endoscopy_UCTN_Code_TTT_1AO_2AG_3AD

## Notes

Endoscopy E-Videos https://eref.thieme.de/e-videos
*E-Videos* is an open access online section of the journal *Endoscopy* , reporting on interesting cases and new techniques in gastroenterological endoscopy. All papers include a high-quality video and are published with a Creative Commons CC-BY license. Endoscopy E-Videos qualify for HINARI discounts and waivers and eligibility is automatically checked during the submission process. We grant 100% waivers to articles whose corresponding authors are based in Group A countries and 50% waivers to those who are based in Group B countries as classified by Research4Life (see: https://www.research4life.org/access/eligibility/ ). This section has its own submission website at https://mc.manuscriptcentral.com/e-videos .
